# Supplementary material for: Pharmacokinetics of Bictegravir in Older Japanese People Living with HIV-1
Source: Microbiol Spectr. 2023 Feb 21;11(2):e05079-22. doi: 10.1128/spectrum.05079-22 (PMC10100687; doi:10.1128/spectrum.05079-22)
Supplement: Supplemental file 1 — Supplemental material. Download spectrum.05079-22-s0001.pdf, PDF file, 0.9 MB [file spectrum.05079-22-s0001.pdf]

Supplementary data 1. PK parameters of each participant

	Cmax (ng/mL)	Tmax (h)	Cmin (ng/mL)	Tmin (h)	Ctrough (ng/mL)	AUC0-24 (h*ng/mL)	T1/2 (h)	CL/F (mL/h)	Vd/F (mL)
Pt.1	21915.2	1.0	5086.0	24.0	5086.0	226631.8	14.7	220.6	4690.4
Pt.2	26348.4	4.0	10003.0	24.0	10003.0	434853.2	16.0	115.0	2649.8
Pt.3	23943.4	3.0	8858.1	24.0	8858.1	326901.8	22.4	153.0	4948.1
Pt.4	29524.6	1.0	14013.7	24.0	14013.7	483346.1	20.5	103.4	3055.6
Pt.5	17559.6	1.0	7481.2	24.0	7481.2	292860.8	16.5	170.7	4056.4
Pt.6	48874.2	3.0	12902.1	0.0	21022.8	682804.1	22.2	73.2	2344.4
Pt.7	45731.1	3.0	15346.7	24.0	15346.7	614344.3	17.2	81.4	2019.3
Pt.8	35433.1	1.0	8918.6	0.0	9151.5	424835.8	14.5	117.7	2470.3
Pt.9	36694.3	3.0	9188.2	24.0	9188.2	455577.4	11.8	109.8	1873.0
Pt.10	8636.7	1.0	2024.6	24.0	2024.6	100249.7	12.6	498.8	9038.7
Mean	29466.1	2.1	9382.2		10217.6	404240.5	16.8	164.4	3714.6
SD	12480.8	1.2	4035.1		5400.7	174590.9	3.8	125.5	2165.3
Geometric Mean	26638.1	1.8	8300.2		8738.0	360543.3	16.5	138.7	3295.1
95% CI Lower	8391.2	0.4	2180.5		2013.3	102061.4	10.0	39.3	1087.2
95% CI Upper	84563.2	7.2	31595.7		37924.3	1273659.3	27.2	489.9	9987.0

Non-compartment analysis by WinNonlin ver.8.2

Supplementary data 2. Correlation between Body weight (A) or renal function (B) and PK parameters.

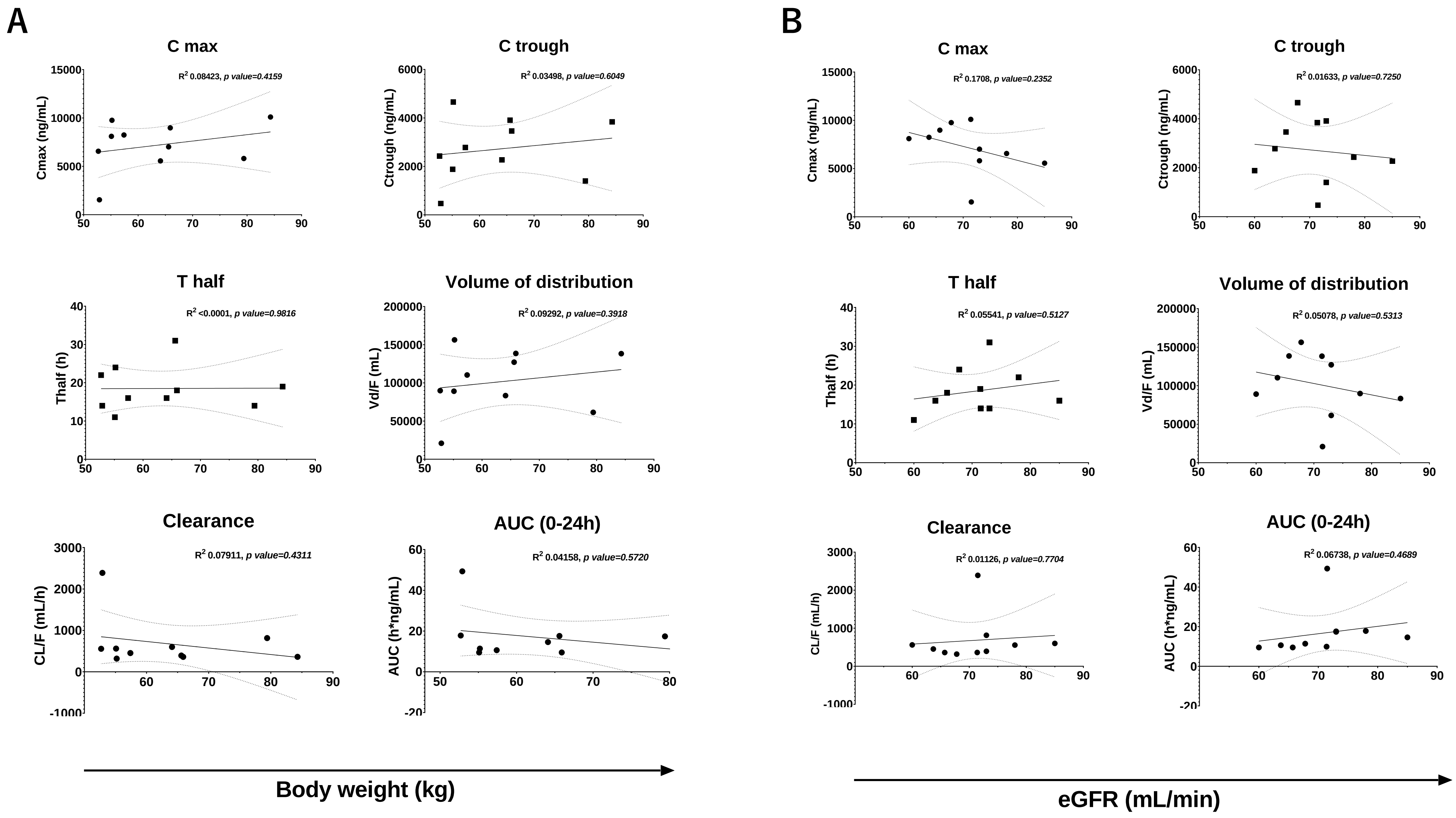

患者登録確認票

ID　　　　　　名前

選択基準

- HIV 感染日本人男性である。　**はい**　いいえ
- 年齢 50 歳以上である。　**はい**　いいえ
- ビクタルビ変更前の薬剤で HIV ウィルス量＜50copies/ml を 3 か月以上維持している（していた）。　**はい**　いいえ
- ビクタルビ開始時にクレアチニンクリアランス 30ｍｌ＞min である（あった）。　**はい**　いいえ

（140－age□□）×　体重　□□.□kg÷（72×血清 Cre□.□□）＝ □□.□ml

- BMI　18 以上 35 未満である。　**はい**　いいえ

体重□□.□kg÷（身長□.□□m×身長□.□□m）＝□□.□

- インテグラーゼ阻害薬の薬剤耐性検出歴がない。　**はい**　いいえ
- インテグラーゼ阻害薬内服中にウィルス学的失敗がなかった。　**はい**　いいえ

（ウィルス学的失敗：内服開始 24 週以降で HIV ウィルス量>200 copies/ml）

- 抗 HIV 薬を朝内服することが出来る。　**はい**　いいえ
- 文書による同意を取得済み。　**はい**　いいえ

除外基準

- ビクタルビ開始時に肝硬変がある（あった）。　はい　**いいえ**
- ビクタルビ開始時に以下の併用禁忌薬を内服している（していた）。　はい　**いいえ**

併用禁忌薬：・リファンピシン・カルバマゼピン・フェノバルビタール
・フェニトイン・ホスフェニトイン・セイヨウオトギリソウ含有食品

- 担当医が本研究への組み入れを不適切と判断した。　はい　**いいえ**

24 時間薬物動態測定を行うサブグループ 10 例における除外基準

- 尿タンパク定性 2 ＋以上　はい　**いいえ**
- ビクタルビと相互作用がある薬剤を内服中である。　はい　**いいえ**

併用注意薬　・ピルジカイニド・リファブチン・アタザナビル・マグネシウム又はアルミニウム含有制酸剤
・鉄剤、カルシウム含有制酸剤（サプリメント等）・メトホルミン
・アシクロビル、バラシクロビル塩酸塩、バルガンシクロビル塩酸塩

20　　　年　　月　　日　　　　担当医師
